# High Glucose Suppresses Human Islet Insulin Biosynthesis by Inducing miR-133a Leading to Decreased Polypyrimidine Tract Binding Protein-Expression

**DOI:** 10.1371/journal.pone.0010843

**Published:** 2010-05-26

**Authors:** Rikard G. Fred, Claus H. Bang-Berthelsen, Thomas Mandrup-Poulsen, Lars G. Grunnet, Nils Welsh

**Affiliations:** 1 Department of Medical Cell Biology, Uppsala University, Uppsala, Sweden; 2 Hagedorn Research Institute, Gentofte, Denmark; 3 Core Unit for Medical Research Methodology, University of Copenhagen, Copenhagen, Denmark; 4 Department of Molecular Medicine and Surgery, Karolinska Institutet, Stockholm, Sweden; University of Kent, United Kingdom

## Abstract

**Background:**

Prolonged periods of high glucose exposure results in human islet dysfunction in vitro. The underlying mechanisms behind this effect of high glucose are, however, unknown. The polypyrimidine tract binding protein (PTB) is required for stabilization of insulin mRNA and the PTB mRNA 3′-UTR contains binding sites for the microRNA molecules miR-133a, miR-124a and miR-146. The aim of this study was therefore to investigate whether high glucose increased the levels of these three miRNAs in association with lower PTB levels and lower insulin biosynthesis rates.

**Methodology/Principal Findings:**

Human islets were cultured for 24 hours in the presence of low (5.6 mM) or high glucose (20 mM). Islets were also exposed to sodium palmitate or the proinflammatory cytokines IL-1β and IFN-γ, since saturated free fatty acids and cytokines also cause islet dysfunction. RNA was then isolated for real-time RT-PCR analysis of miR-133a, miR-124a, miR-146, insulin mRNA and PTB mRNA contents. Insulin biosynthesis rates were determined by radioactive labeling and immunoprecipitation. Synthetic miR-133a precursor and inhibitor were delivered to dispersed islet cells by lipofection, and PTB was analyzed by immunoblotting following culture at low or high glucose. Culture in high glucose resulted in increased islet contents of miR-133a and reduced contents of miR-146. Cytokines increased the contents of miR-146. The insulin and PTB mRNA contents were unaffected by high glucose. However, both PTB protein levels and insulin biosynthesis rates were decreased in response to high glucose. The miR-133a inhibitor prevented the high glucose-induced decrease in PTB and insulin biosynthesis, and the miR-133a precursor decreased PTB levels and insulin biosynthesis similarly to high glucose.

**Conclusion:**

Prolonged high-glucose exposure down-regulates PTB levels and insulin biosynthesis rates in human islets by increasing miR-133a levels. We propose that this mechanism contributes to hyperglycemia-induced beta-cell dysfunction.

## Introduction

Type 2 diabetes is characterized by a decrease in β-cell mass and function either alone or in combination with insulin resistance, which results in an insufficient insulin production and subsequent hyperglycemia [1]–[3]. Glucose is the main stimulator of β-cell function, regulating various cellular processes including insulin gene expression [4], [5], insulin biosynthesis [6]–[8] and insulin secretion [9]. Nevertheless, it has long been known that prolonged exposure to high glucose concentrations results in human islet dysfunction and death [10], a phenomenon known as glucotoxicity [11]. More specifically, culture of human islets for 4 to 9 days resulted in lowered insulin mRNA contents, insulin biosynthesis rates, insulin/proinsulin ratios, glucose-stimulated insulin release and insulin contents [10], [12]. Beta-cell function can also be impaired in response to certain free fatty acids, such as palmitic acid [13], [14], in a process called lipotoxicity [15]. In addition, not only metabolic factors, but also proinflammatory cytokines promote islet dysfunction leading to loss of glucose-stimulated insulin secretion, lowered insulin contents [16], [17], and ultimately, β-cell death through apoptosis [18], [19]. Loss of β-cell mass as a cause of diabetes was long the hallmark of type 1 diabetes, but has in recent years been widely recognized as an important factor in progressive beta-cell dysfunction in type 2 diabetes as well [1], [2], [20].

The relative or absolute insulin deficiency observed in diabetes is in many cases due to a defective insulin biosynthesis insufficient to meet the demand for extended periods of insulin hypersecretion [21], [22]. Several factors are required for controlling the insulin biosynthesis including, but not limited to, transcription of the insulin gene, insulin mRNA stability, translation of the insulin mRNA, and the ability of the endoplasmic reticulum to balance the vast production of proinsulin with the unfolded protein response [23]–[25]. The amount of insulin mRNA available for translation is dependent on both the rate of insulin gene transcription and the stability of the transcript [7], [26], but due to the high copy number of insulin mRNA in the β-cells (app. 50 000 copies/cell at basal conditions), the level of insulin mRNA is controlled mainly by changes in mRNA stability [6], [21].

The regulation of mRNA stability is determined by interactions between regulatory cis-elements in the mRNA molecule and RNA-binding proteins. These interactions are in turn sensitive to many different developmental and environmental stimuli such as cytokines, hormones and nutrients, but also environmental stress such as hypoxia and hyperglycemia [27]. A dominating mediator of increased insulin mRNA stability is the polypyrimidine tract binding protein (PTB). This protein has been shown to induce stability by binding to a conserved sequence in the 3′- untranslated region (UTR) of insulin mRNA [6]. Interestingly it has also been shown that PTB stabilizes mRNA encoding for insulin granule proteins [28], suggesting that PTB stabilizes mRNAs of proteins involved in the entire secretory pathway [29].

MicroRNAs as regulators of protein expression is an evolving research field. By binding to mRNAs, miRNAs can induce translational repression leading to lowered protein levels. It has recently been suggested that certain miRNAs contribute to the pathogenesis of diabetes and may control pancreatic β-cell function [30]. Two of these miRNAs, miR-124a and miR-133a, have recently been shown to target PTB in neuronal and muscle cells, respectively [31], [32]. In addition, using the TargetScan 3.1 software a putative binding site for miR-146 in rat PTB mRNA can be predicted. Interestingly, this miRNA is highly upregulated by cytokines in β-cells [33]. Taken together, at least three different miRNAs may control insulin mRNA translation by regulating PTB levels. The aim of this study was therefore to investigate whether three pathogenetic factors in type 2 diabetes all known to impair beta-cell function in vitro, namely high glucose, sodium palmitate and proinflammatory cytokines, increase miR-124a, miR-133a or miR-146 levels in association with lower PTB levels and lower rates of insulin biosynthesis. We report here that miR-133a is induced by high glucose in human islet cells leading to lower PTB levels.

## Methods

### Islet isolation, culture, and exposure to different beta-cell inhibitory substances

Human pancreatic islets were kindly provided by Prof. Olle Korsgren (Dept. of Radiology, Oncology and Clinical Immunology, Uppsala University Hospital, Uppsala, Sweden), through the Uppsala facility for the isolation of human islets from Scandinavian brain-dead individuals. The human pancreatic islets were isolated from the pancreas of brain-dead organ donors using collagenase digestion and Biocoll gradient centrifugation [34]. After isolation, the islets were cultured free-floating in Sterilin dishes in CMRL 1066 medium containing 5.6 mM glucose, 10% fetal calf serum (FCS), and 2 mM L-glutamine for 1–5 days.

The islet beta-cell percentages determined using Newport Green staining followed by fluorescence microscopy were routinely 30–60%.

To evaluate islet functional quality, batches of twenty islets were perfused with Krebs-Ringer bicarbonate HEPES buffer (KRBH) containing 2 mg/ml human albumin and 1.67 mmol/l glucose at a flow rate of 0.3 ml/min. The glucose concentration was increased to 16.7 mM after a 30-min period. Fractions for insulin measurement were collected every 4 min. For 11 batches of human islets used in different experiments of this study the mean stimulation index in response to glucose was 7.8±2.4.

Following the initial culture period, islets were cultured for an additional 24 hours in CMRL 1066 containing 10% FCS +2 mM glutamine and one of the following supplements: 20 mM glucose; 5.6 mM glucose +0.5 mM sodium palmitate solubilized in 0.5% (weight/volume) fatty acid and lipopolysaccaride free bovine serum albumin (BSA) (Sigma); 20 mM glucose +0.5 mM sodium palmitate solubilized in 0.5% BSA; or 5.6 mM glucose + recombinant human interleukin-1β (IL-1β) (50 units/ml) (PeproTech, London, U.K.) and γ-interferon (IFN-γ) (1,000 units/ml) (PeproTech).

### Real-time RT-PCR analysis

Total islet RNA was purified in Uppsala using the Ultraspec RNA reagent (Biotecx, Houston, TX) according to the instructions of the manufacturer, with the exception that isopropanol-induced RNA precipitation was prolonged at −20 C to ensure efficient miRNA precipitation. All human islet RNA material was sent coded to the Hagedorn Research Institute in Denmark, where the real-time qPCR analyses were performed in a blinded fashion.

#### miRNA

The extracted RNA from human donors was converted to cDNA with the TaqMan microRNA Reverse Transcription kit by the individual RT reaction protocols provided by the manufacturer (Applied Biosystems). For detection of the mature microRNAs, the TaqMan microRNA Assays (Applied Biosystems) were used as described by the manufacturer. The microRNA results were normalized to internal control microRNA (let7c).

#### mRNA

TaqMan Reverse Transcription Reagents and TaqMan Gene Expression Assays were used for production of cDNA and detection by qRT-PCR, respectively, according to manufacturer (Applied Biosystems). The genes of interest were normalized to an internal control (PPIA). Sequences of primers used for semi-quantification of miRNA and mRNA can be provided upon request.

For both types of gene expression the target genes were quantified by relative quantification using the comparative CT method.

### miRNA transfection

Human islets, in groups of 200, were dispersed in 0.5% trypsin for 5 min at 37°C and then treated with DNase I (Amersham Life Science, Piscataway, NJ, USA) (30 units/ml) for 2 min. The resulting islet cell suspensions were placed in non-attachment plates and transfected with the pre-designed miR-133a precursor (Ambion; AM17100), a small, double-stranded and chemically modified RNA molecule designed to mimic the effects of miR-133a, the pre-designed miR-133a inhibitor (Ambion; AM17000), a single stranded and chemically modified RNA-molecule designed to nullify miR-133a activity, or with the negative control miR oligonucleotide (Ambion). To introduce these siRNAs into the cells a liposome reagent, Dharmafect I (Dharmacon), was used. Transfection was carried out according to manufacturers' recommendations using 2 µl Dharmafect and 100 nM of the miRNA oligonucleotides. The transfection was performed in 500 µl serum- and antibiotics-free medium for 2 hours after which the cells were cultured for two days in complete CMRL 1066 medium. The cells were then cultured for another 24 hours at 5.6 or 20 mM glucose before harvest and immunoblot analysis.

### PTB immunoblot analysis

Islet cells were washed once with PBS and then lysed with SDS sample buffer; 2% SDS, 5% β-mercaptoethanol, 100 mM Tris-HCL pH 6.8, 10% glycerol and bromophenolblue. The protein samples were boiled for 5 min before separation on a 9% SDS-PAGE. The separated proteins were electrophoretically transferred to Hybond™-P membranes (Amersham Bioscience), which were then pre-blocked in 2.5% BSA. The membranes were hybridized with monoclonal mouse anti-PTB antibody (Zymed) and monoclonal mouse anti-tubulin antibody (Santa Cruz) for one hour. The horseradish peroxidase conjugated anti-mouse or anti-rabbit antibodies were used as secondary antibodies and luminescence detected with the ECL system (Amersham Bioscience). The resulting bands were then digitalized using the Kodak 4000MM system and the optical densities were measured using the Kodak Molecular Imaging Software.

### Insulin and total protein biosynthesis

Twenty islets in duplicate groups were incubated for 2 h in 100 µl KRBH buffer supplemented with 0.2% bovine serum albumin, 20 mM glucose and 50 µCi/ml L-[3,4,5-^3^H] leucine (GE Healthcare Life Sciences, Chalfont St. Giles, Buckinghamshire, UK). The islets were then washed in Hank's Balanced Salt Solution and homogenized by sonication for 10 seconds in 200 µl of a 50 mM glycine buffer (pH 8.8) supplemented with 0.25% BSA. For insulin analysis, four 10 µl aliquots were mixed with 100 µl 50 mM glycine buffer supplemented with BSA and 0.1% Triton X-100, and in duplicates supplemented with 10 µl of guinea pig anti-human insulin serum (Chemicon International, Temecula, CA, USA) or 10 µl normal guinea pig serum (Harlan Sera-Lab, Loughborough, Leicestershire, UK). One hundred µl protein A-sepharose (GE Healthcare) were added, the tubes tumbled at room temperature for 15 minutes and then washed three times. For total protein biosynthesis, 10 ml aliquots were precipitated with 10% trichloracetic acid. Samples were then solubilized in Ultima Gold scintillation fluid and analyzed by liquid scintillation counting.

## Results

### Effects of high glucose, sodium palmitate or cytokines on miRNA expression in human islets

**Figure 1:**
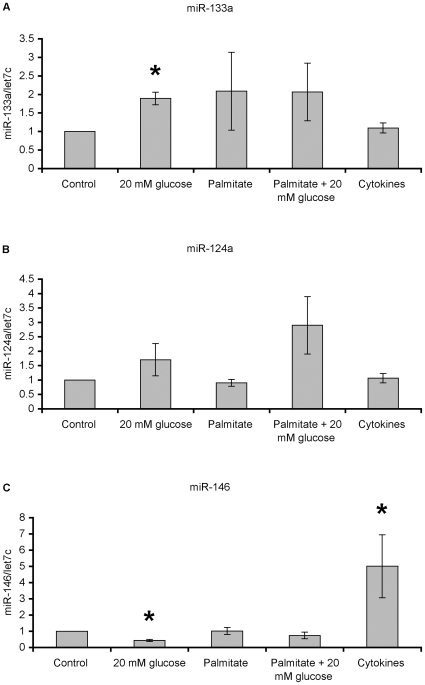
Effects of high glucose, palmitate or cytokines on human islet miR-133a, miR-124a and miR-146 levels. Human islets in groups of 100 were cultured for 24 hours in CMRL 1066 medium with 5.6 mM glucose (control), 20 mM glucose, 5.6 mM glucose and 0.5 mM sodium palmitate solubilized in 0.5% BSA, 20 mM glucose and 0.5 mM sodium palmitate + 0.5% BSA, or 5.6 mM glucose and 50 U/ml IL-1β + 1000 U/ml IFN-γ. The islets were then harvested for RNA isolation, cDNA synthesis and real-time PCR analysis. Primers specific for miR-133a (A), miR-124a (B) and miR-146 (C) were used. Results are expressed per let7c and normalized to control. Results are means ± SEM for 5 independent (islets from 5 different donors) experiments. * denotes p<0.01 using paired Student's t-test when comparing vs. control.

Exposure of islets to high glucose, sodium palmitate or cytokines negatively affects beta-cell function and viability [10], [16], [17], [35] by distinct pathways [36] but in the case of high glucose and cytokines also by partly overlapping pathways [2], [35]. To clarify if the inhibitory effects of these islet stressors were associated with differential expression of specific miRNAs, we analyzed miR-133a, miR-124a and miR-146 levels by semi-quantitative real-time RT-PCR and normalized the results per let7c levels. Let7c is a miRNA that has previously shown no variation in response to high glucose [37] or cytokines (results not shown) in insulin producing cells. Typical thresh-hold cycle numbers for miR-133a, miR-124a and miR-146 were 28–30, 32–34 and 25–27, respectively, indicating a relatively high abundance for miR-146, an intermediary abundance for miR-133a and a low abundance for miR-124a. We observed that a 24h exposure to 20 mM glucose resulted in a doubling of miR-133a levels (Figure 1A). There was also a trend to higher miR-133a levels in response to sodium palmitate (Figure 1A), both at 5.6 and 20 mM glucose, but due to considerable inter donor variation, this effect did not reach statistical significance. A 24h exposure to cytokines did not affect miR-133a levels (Figure 1A).

The same human islet samples were also analyzed for miR-124a levels. In this case, there was only a trend for higher levels of miR-124a in response to high glucose and high glucose+sodium palmitate (Figure 1B). The miR-124a levels were unaltered by sodium palmitate and cytokines (Figure 1B).

Further, we observed a pronounced increase in the levels of miR-146 in response to the cytokines IL-1β and IFN-γ (Figure 1C) in line with previous investigations demonstrating miR-146 induction by cytokines in β-cells [33]. Interestingly, miR-146 levels were significantly decreased in response to high glucose (Figure 1C).

### Effects of high glucose, sodium palmitate or cytokines on PTB mRNA and PTB protein levels in human islets

**Figure 2:**
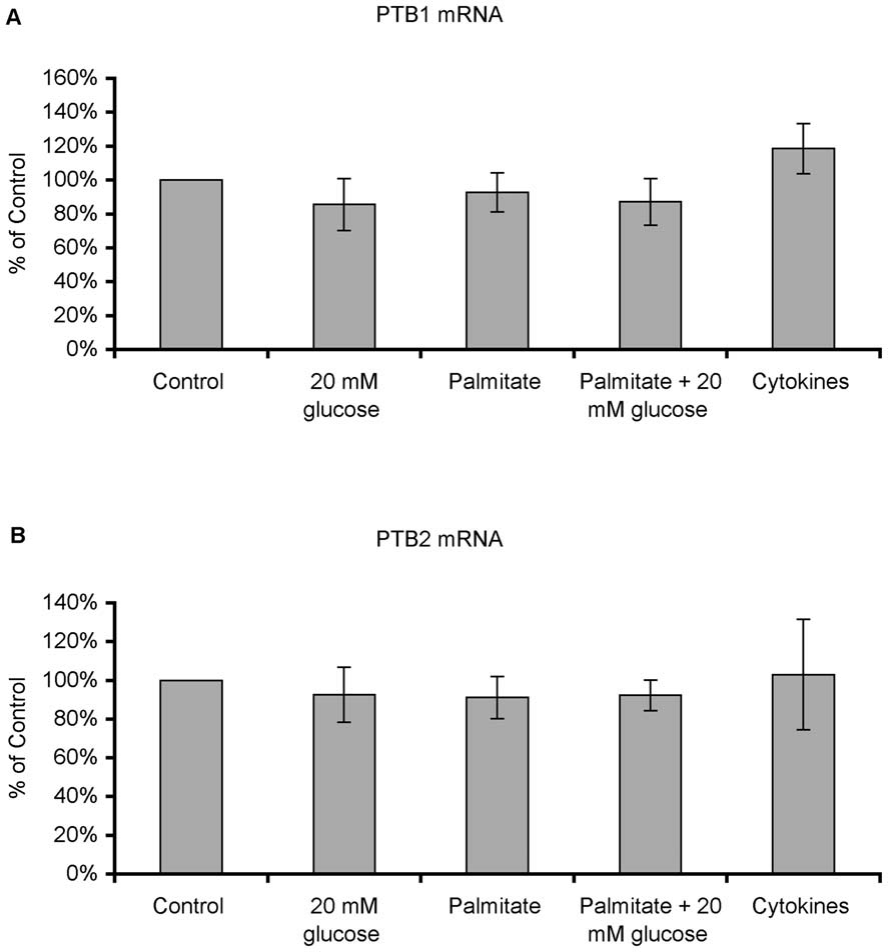
High glucose, palmitate or cytokines do not affect human islet PTB1 mRNA and PTB2 mRNA levels. Human islets were cultured and analyzed by real-time RT-PCR as given in Figure 1. Primers specific for PTB1 (A) and PTB2 (B) were used. Results are means ± SEM for 5 independent experiments.

**Figure 3:**
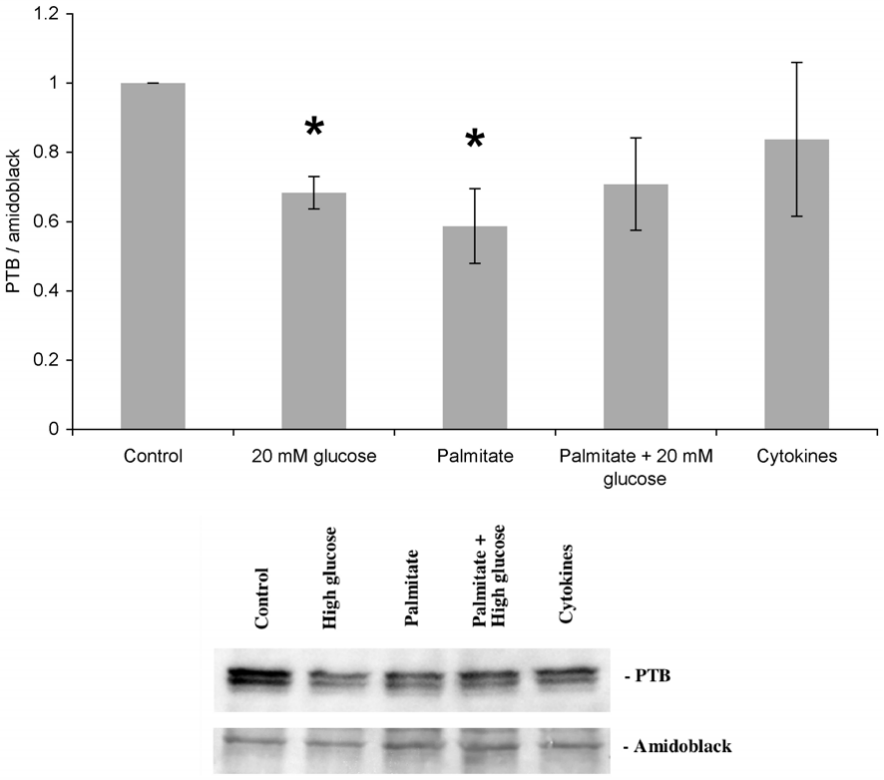
Effects of high glucose, palmitate or cytokines on human islet PTB protein levels. Human islets were cultured as given in Figure 1. Islets were washed and harvested for SDS-PAGE and immunoblotting with anti-PTB antibodies. PTB bands were quantified and normalized to total protein loading using Amidoblack staining. Lower panel shows PTB immunoreactivity and Amidoblack staining of total protein loading from one representative experiment. Results are means ± SEM for 4 independent experiments. * denotes p<0.05 using paired Student's t-test when comparing vs. control.

Having observed that a deleterious concentration of glucose and cytokines increased miR-133a and miR-146, respectively, we next investigated whether PTB mRNA and PTB protein levels were affected. The thresh-hold cycle numbers for PTB1 were 3–4 cycles lower (29 vs. 33 cycles) than those for PTB2, indicating strong expression of PTB1 in human islet cells. None of the experimental conditions altered PTB1- or PTB2 mRNA levels (Figure 2A and 2B). However, PTB protein levels were decreased by 30–40% in islets incubated in the presence of high glucose or sodium palmitate (Figure 3) consistent with the observation that miR-133a inhibits PTB mRNA translation by binding to the 3′-UTR of PTB mRNA [31], [32], and in line with the generally accepted notion that miRNAs often decrease translation without affecting mRNA stability and levels [38], [39]. No effect was observed in response to cytokines (Figure 3), indicating that miR-146, which was strongly induced by cytokines (Figure 1C), does not target PTB-mRNA in human islet cells.

### Effects of high glucose, sodium palmitate or cytokines on insulin mRNA levels and insulin biosynthesis rates

**Figure 4:**
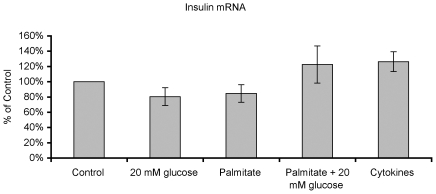
High glucose, palmitate or cytokines do not affect human islet insulin mRNA levels. Human islets were cultured and analyzed by real-time RT-PCR as given in Figure 1. Primers specific for insulin mRNA were used. Results are means ± SEM for 5 independent experiments.

PTB contributes to stabilization of insulin mRNA [6], [28], [40]. To determine whether a high glucose-induced decrease in PTB protein levels affected insulin production, we analyzed insulin mRNA levels and insulin biosynthesis rates. Although there was a trend for lower insulin mRNA levels in islets exposed to high glucose or sodium palmitate, no significant differences were observed (Figure 4). In contrast, insulin biosynthesis rates, when expressed as the percentage insulin biosynthesis of total protein biosynthesis, were decreased by 20% in islets exposed to high glucose for 24h (Table 1). Total protein synthesis rates were not affected by high glucose (Table 1) indicating that a lowered PTB level is associated with impaired insulin production.

**Table 1:** Effects of glucose on human islet insulin biosynthesis.

	5.6 mM Glucose	20 mM Glucose
Insulin Biosynthesis (DPM/islet)	624±58	409±49
Total Protein Biosynthesis (DPM/islet)	12300±1560	9590±1220
Insulin Biosynthesis/Total Protein Biosynthesis (%)	5,3±0,5	4,3±0,4*

Islet in groups of 20 were cultured for 24 hours at 5.6 or 20 mM glucose and then labeled with ^3^H-leucine for two hours in a KRBH buffer containing BSA and 20 mM glucose. Insulin was immunoprecipitated with guinea pig anti-insulin antibodies and protein A sepharose, and total proteins were precipitated with trichloroacetic acid. Incorporated radioactivity was quantified by liquid scintillation counting and results are means ± SEM for 4 observations.

*denotes p<0.05 using paired Student's t-test when comparing vs. control.

### Effects of miR-133a precursor and miR-133a inhibitor on islet PTB protein levels and insulin biosynthesis rates

**Figure 5:**
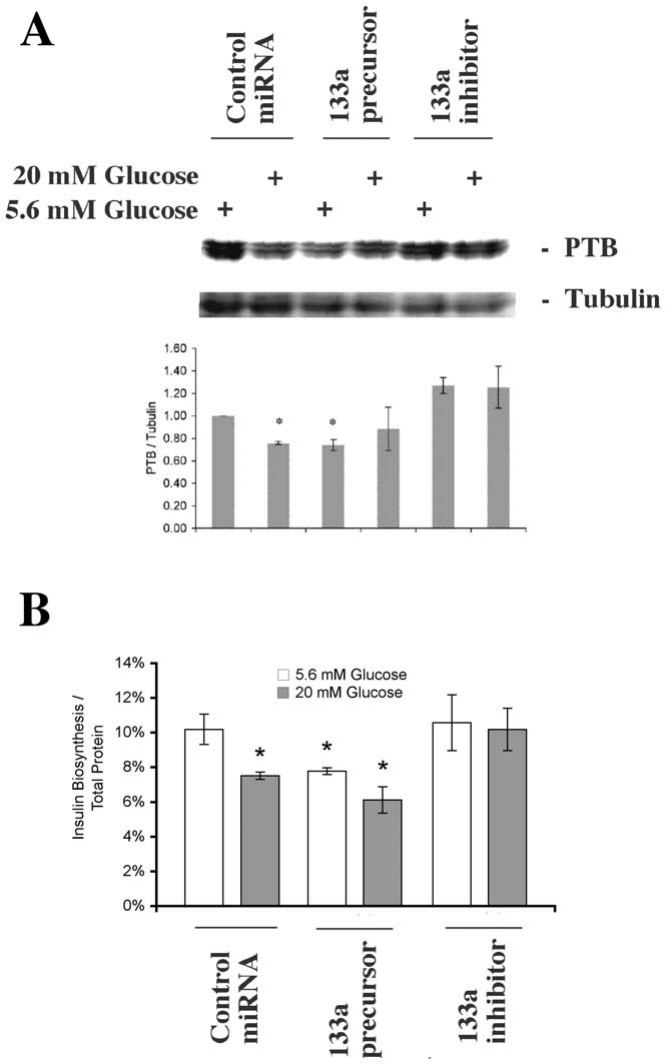
Effects of high glucose, miR-133a precursor or miR-133a inhibitor on human islet PTB protein levels (A) and insulin biosynthesis rates (B). In A, human islet cells were dispersed by trypsin treatment and lipofected with control RNA, miR-133a precursor or miR-133a inhibitor using Dharmafect I. Two days after the lipofection cells were cultured for another 24 hours in either 5.6 or 20 mM glucose. Islets were washed and harvested for SDS-PAGE and immunoblotting with anti-PTB antibodies. Lower panel shows PTB and tubulin immunoreactivity from one representative experiment. In B, human islets were transfected and cultured as in A and insulin biosynthesis rates were determined as in Table 1. Insulin biosynthesis rates were expressed as percentage insulin biosynthesis per total protein biosynthesis. Results are means ± SEM for 3–4 observations. * denotes p<0.05 using paired Student's t-test when comparing vs. control.

To establish that the high glucose-induced increase in miR-133a mediated the decrease in PTB protein levels, we introduced the miR-133a precursor and the miR-133a inhibitor into dispersed islet cells by lipofection. Two days later cells were incubated for 24h at 5.6 or 20 mM glucose and PTB protein levels were determined. Similarly to the experiments of Figure 3, we observed a modest decrease in PTB protein levels in response to the high glucose concentration (Figure 5A). This effect was mimicked by the miR-133a precursor (Figure 5A). The miR-133a inhibitor tended to increase PTB protein levels, both at low and high glucose, but this effect did not reach statistical significance (Figure 5A). In separate control experiments using fluorescein-labeled siRNA as a marker for liposome-mediated uptake of miRNA, transfection efficiency was assessed to approximately 50% (results not shown), which is in line with previous assessments of liposome-mediated islet cell siRNA transfection efficiency [41]. Using the same experimental setup, insulin biosynthesis rates, but not total protein biosynthesis rates, were decrease in human islet cells transfected with the miR-133a precursor, both at low and high glucose concentrations (Table 2 and Figure 5B). The high glucose-induced decrease in insulin biosynthesis was abolished by transfection with the miR-133a inhibitor (Table 2 and Figure 5B).

**Table 2:** Effects of miR-133a precursor and miR-133 inhibitor on human islet insulin biosynthesis.

	5.6 mM Glucose with miR control	20 mM Glucose with miR control	5.6 mM Glucose with miR-133a precursor	20 mM Glucose with miR-133a precursor	5.6 mM Glucose with miR-133a inhibitor	20 mM Glucose with miR-133a inhibitor
Insulin Biosynthesis (DPM/islet)	637±65	410±55*	463±45	335±35*	675±123	504±113
Total Protein Biosynthesis (DPM/islet)	6283±529	5496±808	5970±609	5731±925	6754±1583	5167±1242

Human islets were transfected with miR-133a precursor or inhibitor for two days and then cultured for 24 hours at 5.6 or 20 mM glucose. Insulin and total protein biosynthesis was quantified as given above using 10–20 islet equivalents per group. Results are means ± SEM for 3–4 observations.

*denotes p<0.05 using Student's t-test when comparing vs. control. The percentages insulin biosynthesis per total protein biosynthesis in these experiments are given in Figure 5B.

## Discussion

Here we report that a high glucose concentration increased miR-133a levels and decreased PTB protein levels, paralleled by lowered insulin biosynthesis rates. Since the high glucose effects on PTB protein levels and insulin biosynthesis rates were counteracted by the miR-133a inhibitor, and since the miR-133a precursor mimicked the effects of high glucose, it is likely that miR-133a binds to and inhibits the translation of PTB mRNA in human islet cells, and that high-glucose decreases insulin production, at least in part, by inducing miR-133a. Indeed, miR-133a-induced inhibition of nPTB translation has previously been demonstrated in neuronal cells [31], and it is known that PTB mRNA contains similar miR-133a binding sites as those characterized in nPTB [31]. PTB binds the pyrimidine rich tract of the 3′-UTR of insulin mRNA [6] and other mRNAs coding for secretory proteins present in insulin granules of the beta-cell [28]. A mutation of the PTB-binding site [6], [40] or siRNA-mediated depletion of PTB protein levels [28] results in insulin mRNA destabilization and lower insulin contents. Similarly, in this study a lowered PTB protein level was associated with decreased insulin biosynthesis. Somewhat surprisingly, insulin mRNA levels were not significantly decreased in high glucose cultured islets. A probable explanation for this finding is that high glucose promotes increased insulin gene transcription in human islet cells [42], [43]. Thus, the PTB depletion-induced insulin mRNA destabilization may be counterbalanced by high glucose-induced de novo formation of insulin mRNA. In line with this, it could be speculated that insulin mRNA molecules that do not bind PTB are transferred to a translationally inactive pool or compartment, as observed in other cases [44]. Consequently, cellular PTB levels, rather than total insulin mRNA levels, may determine actual insulin translation rates.

MicroRNA-133a is abundantly expressed in muscle tissue, and insulin-mediated down-regulation of miR-133a levels in human skeletal muscle is attenuated in Type 2 diabetes [45]. In addition, miR-133a contents are increased in cardiomyocytes taken from human diabetic patients [46]. The pathophysiological consequences of such changes in miR-133a levels are unknown, but it has been suggested that uncoupling protein (UCP)-2 is a miR-133a target, and that high miR-133a levels result in a decrease in UCP-2 [47]. UCP-2 is also expressed in islet cells and is activated by reactive oxygen species (ROS), thereby decreasing mitochondrial ROS production [48]. Assuming that miR-133a also targets UCP-2 in islet cells, high glucose-induced increase in miR-133a may result in lower UCP-2 activity and higher ROS production, contributing to glucotoxicity. Further studies on the consequences of miR-133a-mediated reduction on UCP-2 levels in human islet cells are warranted.

MiR-124a is highly expressed and targets PTB in neuronal cells [32]. In insulin producing cells miR-124a may directly target the secretory vesicle protein Rab27A [49] and the transcription factor FoxA2 [50], possibly leading to alterations in beta-cell differentiation, glucose metabolism and insulin release. A high glucose concentration upregulates mir-124a in a mouse beta-cell line [37]. In the present study, a high glucose concentration induced miR-124a only in islet from some of the donors. Also the islet miR-133a response to sodium palmitate was highly inconsistent from one donor to another. The reason for this inter-donor variability is not clear, but variability in cellular composition and metabolic or functional status might have influence the response to high glucose and sodium palmitate. Nevertheless, in previous studies over-expression of miR-124a in insulin producing cells resulted in perturbations in calcium levels and glucose-induced insulin release [49], [50], indicating a negative impact of an increased level of miR-124a on islet cell function.

MicroRNA-146 levels increase in response to cytokines in rodent beta-cells and human islets [33] and to sodium palmitate in rodent beta-cells [49]. These effects of cytokines were confirmed by our data, whereas the effects of sodium palmitate were at variance with the present investigation. The reason for this discrepancy is unclear, but it may be that human and rodent islet cells react differently to sodium palmitate. Cytokine-induced miR-146 induction mediates feed back inhibition of interleukin-1 receptor- and Toll-like receptor-induced signaling by promoting down-regulation of IRAK and TRAF6 [33]. Cytokine-induced up-regulation of miR-146 has been suggested to enhance beta-cell destruction [33], but it is also possible, at least in theory, that miR-146 induction and IRAK + TRAF6 down-regulation leads to increased resistance to cytokines.

In the present investigation, human islets responded with a markedly decreased miR-146 level in response to a high glucose concentration. This finding could be of considerable importance to our understanding of Type 2 diabetes. Hyperglycemia has been suggested to mediate islet inflammation and beta-cell dysfunction in Type 2 diabetes by increasing islet interleukin-1 production [2]. Our finding points to the novel possibility that high glucose concentrations increase islet sensitivity to interleukin-1- and Toll-like receptor activation by counteracting miR-146-induced down-regulation of IRAK and TRAF6. Further studies in the field of miRNA-mediated control of islet cytokine-signaling are needed to clarify these questions.

In conclusion, we report for the first time the existence of a miRNA-regulated mechanism in human islet cells that mediates the suppressive effects of high glucose on insulin biosynthesis. The impact of the high glucose-induced increase in miR-133a on insulin biosynthesis rates was not dramatic, but this mechanism may act in synergy with other high glucose-induced suppressive mechanisms leading to beta-cell failure and aggravation of Type 2 diabetes. Finally it is possible that other miRNA-controlled mechanisms participate in high glucose-induced beta-cell dysfunction. For example, miR-146-mediated control of cytokine-receptor-signaling events may regulate beta-cell sensitivity to inflammatory factors, and miR-133a targeting of UCP-2 may control mitochondrial output of ROS.

## References

[1] Cnop M, Welsh N, Jonas JC, Jörns A, Lenzen S (2005). Mechanisms of pancreatic beta-cell death in Type 1 and Type 2 diabetes: many differences, few similarities.. Diabetes.

[2] Donath MY, Størling J, Berchtold LA, Billestrup N, Mandrup-Poulsen T (2008). Cytokines and β-cell Biology: from Concept tp Clinical Translation.. Endocr Rev.

[3] Tish R, McDevitt H (1996). Insulin-dependent diabetes mellitus.. Cell.

[4] Melloul D, Ben-Neriah Y, Cerasi E (1993). Glucose modulates the binding of an islet-specific factor to a conserved sequence within the rat I and the human insulin promoters.. Proc Natl Acad Sci U S A.

[5] Ohneda K, Mirmira RG, Wang J, Johnson JD, German MS (2000). The homeodomain of PDX-1 mediates multiple protein-protein interactions in the formation of a transcriptional activation complex on the insulin promoter.. Mol Cell Biol.

[6] Tillmar L, Carlsson C, Welsh N (2002). Control of insulin mRNA stability in rat pancreatic islets. Regulatory role of the 3′-untranslated region pyrimidine-rich sequence.. J Biol Chem.

[7] Welsh M, Nielsen DA, MacKrell AJ, Steiner DF (1985). Control of insulin gene expression in pancreatic β-cells and in an insulin producing cell line, RIN-5F cells.. J Biol Chem.

[8] Wicksteed B, Herbert TP, Alacon C, Lingohr MK, Moss LG (2001). Cooperativity between the preproinsulin mRNA untranslated regions is necessary for glucose-stimulated translation.. J Biol Chem.

[9] Ashcroft FM, Rorsman P (1989). Electrophysiology of the pancreatic beta-cell.. Prog Biophys Mol Biol.

[10] Eizirik DL, Korbutt GS, Hellerström C (1992). Prolonged exposure of human pancreatic islets to high glucose concentrations in vitro impairs the β-cell function.. J Clin Invest.

[11] Robertson RP, Zhang HJ, Pyzdrowski KL, Walseth TF (1992). Preservation of insulin mRNA levels and insulin secretion in HIT cells by avoidance of chronic exposure to high glucose concentrations.. J Clin Invest.

[12] Marshak S, Leibowitz G, Bertuzzi F, Socci C, Kaiser N (1999). Impaired beta-cell functions induced by chronic exposure of cultured human pancreatic islets to high glucose.. Diabetes.

[13] Lupi R, Del Guerra S, Marselli L, Bugliani M, Boggi U (2004). Rosiglitazone prevents the impairment of human islet function induced by fatty acids: evidence for a role of PPARgamma2 in the modulation of insulin secretion.. Am J Physiol Endocrinol Metab.

[14] Zhou YP, Grill V (1995). Long term exposure to fatty acids and ketones inhibits B-cell functions in human pancreatic islets of Langerhans.. J Clin Endocrinol Metab.

[15] Unger RH (1995). Lipotoxicity in the pathogenesis of obesity-dependent NIDDM. Genetic and clinical implications.. Diabetes.

[16] Eizirik DL, Sandler S, Welsh N, Cetkovic-Cvrlje M, Nieman A (1994). Cytokines suppress human islet function irrespective of their effects on nitric oxide generation.. J Clin Invest.

[17] Mandrup-Poulsen T, Bendtzen K, Nielsen JH, Bendixen G, Nerup J (1985). Cytokines cause functional and structural damage to isolated islets of Langerhans.. Allergy.

[18] Eizirik DL, Mandrup-Poulsen T (2001). A choice of death: the signal –transduction of immune-mediated β-cell apoptosis.. Diabetologia.

[19] Rabinovitch A, Suarez-Pinzon W, Strynadka K, Ju Q, Edelstein D (1999). Transfection of human pancreatic islets with an anti-apoptotic gene (bcl-2) protects beta-cells from cytokine-induced destruction.. Diabetes.

[20] Butler AE, Janson J, Bonner-Weir S, Ritzel R, Rizza RA (2003). Beta-cell deficit and increased beta-cell apoptosis in humans with Type 2 diabetes.. Diabetes.

[21] Fred RG, Welsh N (2009). The importance of RNA binding proteins in preproinsulin mRNA stability.. Mol Cell Endocrinol.

[22] Ozcan U, Yilmaz E, Ozcan L, Furuhashi M, Vaillancourt E (2006). Chemical chaperones reduce ER stress and restore glucose homeostasis in a mouse model of Type 2 diabetes.. Science.

[23] Hay CW, Docherty K (2006). Comparative analysis of insulin gene promoters: implications for diabetes research.. Diabetes.

[24] Kaneto H, Kawamori D, Matsuoka TA, Kajimoto Y, Yamasaki Y (2005). Oxidative stress and pancreatic beta-cell dysfunction.. Am J Ther.

[25] Minn AH, Lan H, Rabaglia ME, Harlan DM, Peculis BA (2005). Increased insulin translation from an insulin splice-variant overexpressed in diabetes, obesity, and insulin resistance.. Mol Endocrinol.

[26] Nielsen DA, Welsh M, Casadaban MJ, Steiner DF (1985). Control of insulin gene expression in pancreatic beta-cells and in an insulin-producing cell line, RIN-5F cells. I. Effects of glucose and cyclic AMP on the transcription of insulin mRNA.. J Biol Chem.

[27] Guhaniyogi J, Brewer G (2001). Regulation of mRNA stability in mammalian cells.. Gene.

[28] Knoch KP, Bergert H, Borgonovo B, Saeger HD, Altkrüger A (2004). Polypyrimidine tract-binding protein promotes insulin secretory granule biogenesis.. Nat Cell Biol.

[29] Gama-Carvalho M, Barbosa-Morais NL, Brodsky AS, Silver PA, Carmo-Fonseca M (2006). Genome-wide identification of functionally distinct subsets of cellular mRNAs associated with two nucleocytoplasmic-shuttling mammalian splicing factors.. Genome Biol.

[30] Kolfschoten IG, Roggli E, Nesca V, Regazzi R (2009). Role and therapeutic potential of microRNAs in diabetes.. Diabetes Obes Metab.

[31] Boutz PL, Chawla G, Stoilov P, Black DL (2007). MicroRNAs regulate the expression of the alternative splicing factor nPTB during muscle development.. Genes Dev.

[32] Makeyev EV, Zhang J, Carrasco MA, Maniatis T (2007). The microRNA miR-124 promotes neuronal differentiation by triggering brain-specific alternative pre-mRNA splicing.. Mol Cell.

[33] Roggli E, Britan A, Gattesco S, Lin-Marq N, Abderrahmani A (2010). Involvement of microRNAs in the cytotoxic effects exerted by proinflammatory cytokines on pancreatic {beta}-cells.. Diabetes.

[34] Goto T, Tanioka Y, Sakai T, Matsumoto I, Kakinoki K (2005). Successful islet transplantation from a single pancreas harvested from a young, low-BMI, non-heart-beating cadaver.. Transplant Proc.

[35] Zhou R, Tardivel A, Thorens B, Choi I, Jürg Tschopp J (2009). Thioredoxin-interacting protein links oxidative stress to inflammasome activation.. Nat Immunol.

[36] Akerfeldt MC, Howes J, Chan JY, Stevens VA, Boubenna N (2008). Cytokine-induced beta-cell death is independent of endoplasmic reticulum stress signaling.. Diabetes.

[37] Tang X, Muniappan L, Tang G, Ozcan S (2009). Identification of glucose-regulated miRNAs from pancreatic {beta} cells reveals a role for miR-30d in insulin transcription.. RNA.

[38] Jackson RJ, Hellen CU, Pestova TV (2010). The mechanism of eukaryotic translation initiation and principles of its regulation.. Nat Rev Mol Cell Biol.

[39] Selbach M, Schwanhäusser B, Thierfelder N, Fang Z, Khanin R (2008). Widespread changes in protein synthesis induced by microRNAs.. Nature.

[40] Tillmar L, Welsh N (2004). Glucose-induced binding of the polypyrimidine tract-binding protein (PTB) to the 3′-untranslated region of the insulin mRNA (ins-PRS) is inhibited by rapamycin.. Mol Cell Biochem.

[41] Hägerkvist R, Mokhtari D, Myers JW, Tengholm A, Welsh N (2005). siRNA produced by recombinant dicer mediates efficient gene silencing in islet cells.. Ann N Y Acad Sci.

[42] Evans-Molina C, Garmey JC, Ketchum R, Brayman KL, Deng S (2007). Glucose regulation of insulin gene transcription and pre-mRNA processing in human islets.. Diabetes.

[43] Muller D, Huang GC, Amiel S, Jones PM, Persaud SJ (2006). Identification of insulin signaling elements in human β-cells: Autocrine regulation of insulin gene expression.. Diabetes.

[44] Zhang J, Bahi N, Llovera M, Comella JX, Sanchis D (2009). Polypyrimidine tract binding proteins (PTB) regulate the expression of apoptotic genes and susceptibility to caspase-dependent apoptosis in differentiating cardiomyocytes.. Cell Death Differ.

[45] Granjon A, Gustin MP, Rieusset J, Lefai E, Meugnier E (2009). The microRNA signature in response to insulin reveals its implication in the transcriptional action of insulin in human skeletal muscle and the role of a sterol regulatory element-binding protein-1c/myocyte enhancer factor 2C pathway.. Diabetes.

[46] Xiao J, Luo X, Lin H, Zhang Y, Lu Y (2007). MicroRNA miR-133 represses HERG K+ channel expression contributing to QT prolongation in diabetic hearts.. J Biol Chem.

[47] Chen X, Wang K, Chen J, Guo J, Yin Y (2009). In vitro evidence suggests that miR-133a-mediated regulation of uncoupling protein 2 (UCP2) is an indispensable step in myogenic differentiation.. J Biol Chem.

[48] Mehta SL, Li PA (2009). Neuroprotective role of mitochondrial uncoupling protein 2 in cerebral stroke.. J Cereb Blood Flow Metab.

[49] Lovis P, Gattesco S, Regazzi R (2008). Regulation of the expression of components of the exocytotic machinery of insulin-secreting cells by microRNAs.. Biol Chem.

[50] Baroukh N, Ravier MA, Loder MK, Hill EV, Bounacer A (2007). MicroRNA-124a regulates Foxa2 expression and intracellular signaling in pancreatic beta-cell lines.. J Biol Chem.

